# Bathing trunk nevus

**DOI:** 10.4103/0971-9261.55160

**Published:** 2009

**Authors:** A. Y. Kshirsagar, K. S. Shukla, Y. P. Nikam, R. B. Garg, T. U. Sholapurkar

**Affiliations:** Department of Surgery, Krishna Institute of Medical Sciences, University, Karad, India

**Keywords:** Bathing trunk nevus, congenital nevomelanocytic nevus, congenital pigmented nevi

**Figure 1:**
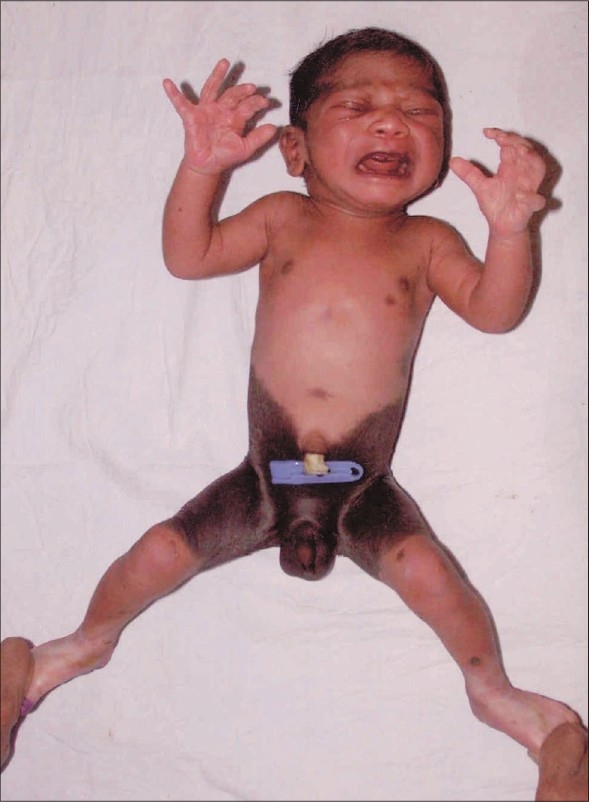
Anterior view of child with costume hairy nevus

We report a one-day-old male child born with bathing trunk hairy nevus [Figure 1]. Congenital hairy nevus denotes a pigmented surface lesion present at birth in one per cent of newborns, but incidence of giant congenital pigmented nevi is less than 1 in 20,000 births. These nevi are significant because of association with leptomeningeal melanocytosis or neurofibromatosis and predispose to malignant melanoma. The exact cause is unknown. There may be hereditary factors, autosomal dominance or other multifactor determinants. Management depends upon size, location and malignant transformation. Surgical excision is the mainstay of treatment. Cultured epidermal autograph has been used successfully to cover postoperative large surface area defect.

